# Drinking Water

**Published:** 1874-04

**Authors:** 


### DRINKING WATER.

THERE is very little water used for drinking purposes in Great Britain, France, Austria and Germany, in the belief that it is not healthful. In a certain part of our own country, within five miles of New York City, a spring at the bottom of a hill, which sent out its cool, and clear, and bubbling drops, fairly sparkling in their apparent purity, gave a whole family typhoid fever. On letting the water stand a sediment was observed at the bottom, and, being examined with a microscope, the dregs were found to be those of a privy a little distance up the hill-side. Hence, families moving to new places should test the water which is to be used for cooking and drinking, thus: put half a pint of the water into a perfectly clean and clear glass bottle with a ground glass stopper, and add a level teaspoonful of the purest loaf sugar. If the water does not become turbid, cloudy, within ten days, then it is certain that there is no sewage, no drain from privies in it. But there may be other impurities, metallic or otherwise insoluble, not subject to fermentation arising from the addition of sugar to a liquid containing sewage dregs; hence the sugar test is deceptive except as to pointing out a particular kind of impurity. There would be no safety in relying upon it as a sure means of designating pure water.

A better way is to decide whether there are any organic impurities, thus: place some of the water in a clean, clear glass bottle with a ground stopper, in the sun for a few days, or where the thermometer is not lower than 70° F. If it does not become offensive to the smell in three or four days, it may be considered free from organic impurities. If the liquid contains insoluble mineral matters they will settle at the bottom in a few days and can be seen, and also, if there are insoluble vegetable or animal substances; in either case, if the water must be used, it should first be filtered through charcoal and oxide of iron, which detains and destroys all the organic matter in any liquid. All wells and springs of drinking water should be thoroughly cleaned and washed out in October of every year, because the first rains of autumn, after the drought of summer, in sinking into the earth and finding its way into wells, carries with it those miasmatic matters which abound at that time of the year, and the use of which water is followed by the very effects, during the winter, that the breathing of the air which contained them did in the later summer and earlier autumn.

Hereafter, as our country grows older, there may be so many sources of deleterious contamination that an impression may be made and extended that it is unwholesome to drink water, and the next step will be, as in Great Britain, Germany and France, that porter, and beer, and wine, and other forms of spirits will take the place of water. The death rate of New Orleans, with all its disadvantages of luxuriant vegetation and almost tropical sun, and its level site, making drainage almost impossible, is twenty-nine out of a thousand of the population yearly. In New York City it is thirty-two. In New York we use the sparkling Croton. In New Orleans, where we lived many years, and on the sugar and cotton plantations, the water used for cooking, drinking and washing is obtained from cisterns above ground, holding a thousand or two or more gallons. These cisterns are simply immense wooden barrels or tanks, placed on the shady side of the house, or under a shed with a top, beside the cover of the tank, into which the water comes from the roof of the house. When the cisterns are large the water is palatable, even in midsummer, without ice. Considering that all farm houses have so many stables, barns, pens, out houses and drains about them, it may well be inquired if the general health of farmers' families would not be improved if above ground cistern water was exclusively used for drinking and culinary purposes. It is the almost universal belief of those who live on the banks of the Missisippi that its waters are the healthiest in the world—and yet they are so turbid that if poured into the clearest glass tumbler or bottle it is impossible to see through it, yet if allowed to stand still it settles and becomes quite clear in a few hours. But we have cleared it many times in five minutes by tying a piece of alum to a thread, suspending it in the water and imparting a circular motion to it. The alum combines with the solid materials and all fall to the bottom, leaving no taste of alum whatever in the water.

